# Seasonal variation of *Salmonella enterica* prevalence in milk and cottage cheese along the dairy value chain in three regions of Ethiopia

**DOI:** 10.1186/s40550-024-00108-4

**Published:** 2024-05-10

**Authors:** Henok Nahusenay Admasu, Abdi Bedassa, Tesfaye Sisay Tessema, Jasna Kovac, Jessie L. Vipham, Ashagrie Zewdu Woldegiorgis

**Affiliations:** 1 Head quarter Food science and Nutrition research directorate, Ethiopian Institute of Agricultural Research, PO Box 2003, Addis Ababa, Ethiopia; 2 Ethiopian Institute of Agricultural Research, National Agricultural Biotechnology Research Center, PO Box 249, Holeta, Ethiopia; 3 Institute of Biotechnology, Addis Ababa University, New Graduate Building, P.O. Box 1176, Addis Ababa, Ethiopia; 4 Department of Food Science, The Pennsylvania State University, 437 Erickson Food Science Building, University Park, Pennsylvania, PA 16802 USA; 5 Department of Animal Sciences and Industry, Kansas State University, 247 Weber Hall, Manhattan, KS 66506 USA; 6 Center for Food Science and Nutrition, College of Natural Sciences, Addis Ababa University, New Graduate Building, P.O. Box 1176, Addis Ababa, Ethiopia

**Keywords:** Cottage cheese, Raw milk, Pasteurized milk, Season, *Salmonella enterica*, *inv*A gene, Ethiopia

## Abstract

Seasonal fluctuations influence foodborne illness transmission and affect patterns of microbial contamination of food. Previous investigations on the seasonality of *Salmonella enterica* prevalence in dairy products in Ethiopia have been minimal. However, such data are needed to inform strategic development of effective interventions to improve food safety, as seasonal differences may affect intervention strategies. This study was conducted to identify differences in the prevalence of *Salmonella* in milk and cheese samples between wet and dry seasons. A longitudinal study design was utilized with a random sampling occurring during both dry and wet seasons. A total of 448 milk and cottage cheese samples were collected from Oromia, Sidama, and Amhara regions. Samples were tested for *Salmonella* using the ISO 6579-1: 2008 method, followed by PCR confirmation. A chi-square test was conducted to assess the significance of differences in the prevalence of *Salmonella* in the samples between the two seasons. Results from this study showed a higher prevalence of *Salmonella* in all sample types during the dry season (*P* < 0.05). Moreover, when comparing raw milk, pasteurized milk, and cottage cheese samples, a significant difference was observed in *Salmonella* prevalence from raw milk samples (27.08%) collected in the Oromia region. Additionally, data showed a significantly higher prevalence of *Salmonella* in samples collected from raw milk producers (29.17%) during the wet season (*P* < 0.05). This study indicates that in order to enhance the safety of dairy products in Ethiopia, comprehensive, long-term awareness building on hygienic milk production and handling that consider seasonal influence is warranted.

**Supplementary Information:**

The online version contains supplementary material available at 10.1186/s40550-024-00108-4.

## Introduction

Foodborne illnesses continue to have significant adverse effects on global public health, the economy, and society (WHO, 2021). More than 600 million cases and 420, 000 deaths occur each year, with the proportion of this burden being predominately higher in low- and middle-income countries (Havelaar et al. 2015). In Ethiopia alone, around 60% of disease what type of diseases and in which population emerged from production and processing of perishable foods like dairy products (Wabto et al., 2017).

Milk and its products can be contaminated by pathogenic bacteria before it leaves the farm, mainly as a result of environmental contamination through air, water, barn, feed, and pasture during production and processing, (Lucey 2015; Tegegne and Tesfaye 2017). Thus, this will contribute to the acquisition of these bacteria by humans through consumption (Chen et al., 2018). In addition, direct passage from the blood (of cow) into milk (systemic infection), mastitis (udder infection), and fecal contamination (external contamination of milk from the environment during or after milking) are the main routes for the introduction of pathogenic microbes to milk (Lucey 2015; Belina et al., 2021). Most pathogenic bacteria are effectively controlled by milk pasteurization. However, inadequate pasteurization of milk results in human exposure to pathogenic bacteria in the pasteurized milk (Cancion-Padilla et al., 2017).

*Salmonella eneterica* is a foodborne pathogen with over 2500 serotypes, with more than 1540 belonging to *the Salmonella enterica* subspecies *enterica*; which accounts for the majority of *Salmonella* infections in humans (Eng et al. 2015). *Salmonella* is the leading cause of bacterial foodborne illness in the world (Keba et al. 2020; Pal et al. 2020). Foods contaminated with *Salmonella*, notably raw milk and its products, are responsible for an estimated 2,458,000 cases and 4,100 annual deaths in Africa (Majowicz et al., 2010; Pal et al. 2020). Furthermore, *Salmonella* has been linked to bacteremia in immunocompromised people, infants, and new babies in Africa, and dairy animals have been identified as primary sources of salmonellosis in humans (Feasey et al. 2012; Nyenje and Ndip., 2013).

Many infectious diseases exhibit seasonality, meaning that the prevalence of infections can vary due to seasonal weather patterns (Naumova et al. 2007). This is likely due to seasonal variation in temperature and precipitation, which result in seasonal prevalence peaks that are interspersed with low levels of infections (Green et al. 2006; Lal et al. 2012). Statistics show a correlation between short-term temperature variations and foodborne illnesses or infections (Ebi 2011; Semenza et al. 2012). Long-term climatic changes, such as elevated average air temperatures and increased precipitation frequency or intensity, have been shown to have an impact on short-term changes (Smith et al. 2019). Within certain bounds, the survival rates of most enteric pathogens in the environment are positively associated with ambient temperature (Semenza et al. 2012). Due to environmental influences such as seasonal weather and climate fluctuations, the patterns of pathogen prevalence consequently change (Semenza et al. 2012). Notably, *Salmonella* and *Campylobacter infections* have been shown to peak in the summer with demonstrated seasonal tendencies (Kovats et al. 2004; Rivero et al. 2012).

Environmental change, most notably season, weather, and climate, can also influence foodborne illness transmission indirectly by altering food consumption behaviors, livestock susceptibility to pathogens, and vector transmission due to range expansion, increased activity, and reproduction rates (Séguin et al., 2008; Smith et al. 2019). When livestock are stressed by temperature changes, pathogenic enteric bacteria, such as Salmonella, can colonize animals more readily (Keen et al. 2003; Pangloli et al. 2008). If processing hygiene standards are breached, this can raise the risk of fecal contamination of animal-based foods, such as raw milk and meat (Williams et al. 2015).

The relationship between temperature and precipitation and the frequency of salmonellosis cases is supported in multiple scientific publications (Rose et al. 2001; Zhang et al. 2010; Putturu et al. 2015; Stephen and Barnett 2016; Park et al. 2018; Judd et al. 2019; Ung et al. 2019). Recently, Bedassa et al. (2023) reported a 13.8% prevalence of the organism in milk and cheese in samples collected from the Oromia, Sidama, and Amhara regions of the country in the dry season. Studies on seasonal variations and regional distributions of pathogenic organisms are crucial for developing strategic interventions and anticipating outbreaks.

This research was planned with the intention of assessing whether seasonal variation affected the amount of *Salmonella enterica* found in milk and cottage cheese samples that were taken from study locations in the Amhara, Sidma, and Oromia regions of Ethiopia throughout the dry and wet seasons.

## Materials and methods

### Study area and study design

To determine the prevalence of *Salmonella* raw milk, pasteurized milk, and cottage cheese samples, a longitudinal study design was utilized in three important milk sheds in Ethiopia i.e. Deber-Zeit (Oromia), Hawassa (Sidama), and Bahir Dar (Amhara). The rainy (wet) and warm (dry) seasons of the nation’s climate profile were taken into consideration when designing a sampling plan to capture the seasonal differences. The selection criteria were based on the previously published data on prevalence of *Salmonella* in dry season (Bedassa et al.,2023), which showed that samples taken from these sites had a higher prevalence of *Salmonella* than those taken from the other study sites, most notably Hawassa City. Second, the selected study locations had a complete set of value chain actors, including producers, collectors, processors, and retailers. Due to the region’s recent reorganization, the SNNP region that was referenced in our previous report (Bedassa et al. 2023) was now the Sidama region.

### Sample collection

In the wet season (June, July, and August), a total of 228 cow milk and cottage cheese samples (92 raw milk, 92 pasteurized milks, and 44 cottage cheese) were collected from Debre Zeit (*n* = 120), Hawassa (*n* = 60), and Bahir Dar (*n* = 48). Previously published data from the dry season was used for the seasonal comparison (Bedassa et al. 2023). The dry season samples were collected from the same producers, collectors, processors, and retailers as during wet season (January, February, March, and April). Dairy food samples were collected aseptically using sterile containers and transported by a portable refrigerator, which was maintained at 4 ℃, to the Holeta Microbial Biotechnology Laboratory of the National Agricultural Biotechnology Research Center (NABRC) for microbial analysis. Microbiological analyses were conducted within 12 h from sample collection.

### Detection and conformation of S. Enterica

**Fig. 1:**
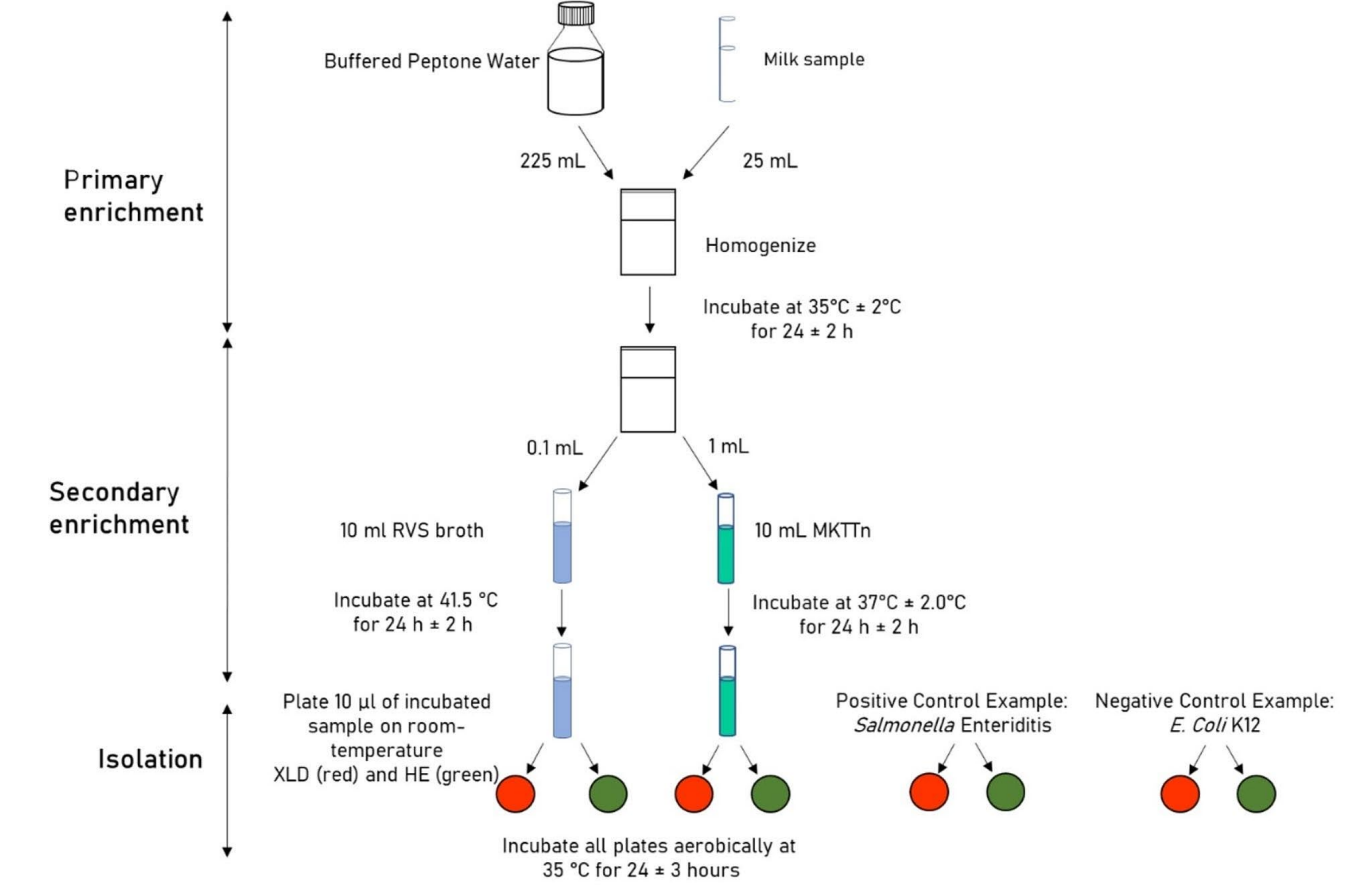
Overall isolation procedure of *S. enterica*, demonstrates the protocol starting from enrichment to selective plating (Source Ensure Dairy Project)

Figure 1 illustrates *Salmonella* enrichment and isolation according to ISO 6579-1: (ISO 2017). The full detailed procedure of *Salmonella* enrichment and isolation were outlined in our prior report (Bedassa et al. 2023). Buffered Peptone Water (BPW) (Oxoid, CM 0509) was used for the pre-enrichment of Salmonella, and it was incubated for 18 h at 35 °C. Salmonella was selectively enriched using sterile Muller Kaufmann Tetrathionate (MKTTn) broth (HiMedia) and Rappaport Vassiliadis (RV) broth (HiMedia), respectively. An inoculated RV broth was incubated at 41 °C for 24 h, while an inoculated MKTTn broth was incubated at 37 °C. Selective enriched presumed *Salmonella* were plated on Xylose Lysine Deoxycholate (XLD) agar and Hektoen Enteric (HE) agar (HiMedia). For 24 h, inoculated plates were incubated aerobically at 37 °C. Presumptive isolates were further subcultured onto Brain Heat Infusion (BHI) agar and incubated at 35 °C for 24 h for molecular conformation. *Salmonella* isolates were confirmed utilizing PCR targeting the *invA* gene (Galán and Curtiss III, 1991). The full and detailed procedure of *Salmonella* PCR conformation was outlined in our prior report (Bedassa et al. 2023). Each electrophoresis run included a 100 bp DNA ladder, as well as positive (*Salmonella enterica ATTCC* 35,664) and negative controls (nuclease free water).

### Statistical analysis

Descriptive statistics of the prevalences were performed using Microsoft Excel, and a chi-square test was performed using SPSS version 20.0 software to evaluate the statistical significance of variations in *Salmonella enterica* prevalence among sample types (raw and pasteurized milk, cottage cheese), seasons, and across the diary value chain. *P* < 0.05 was used to determine statistical significance for differences.

## Results

### Comparison of overall prevalence of *S. Enterica between* dry and wet seasons across the sample type

**Fig. 2:**
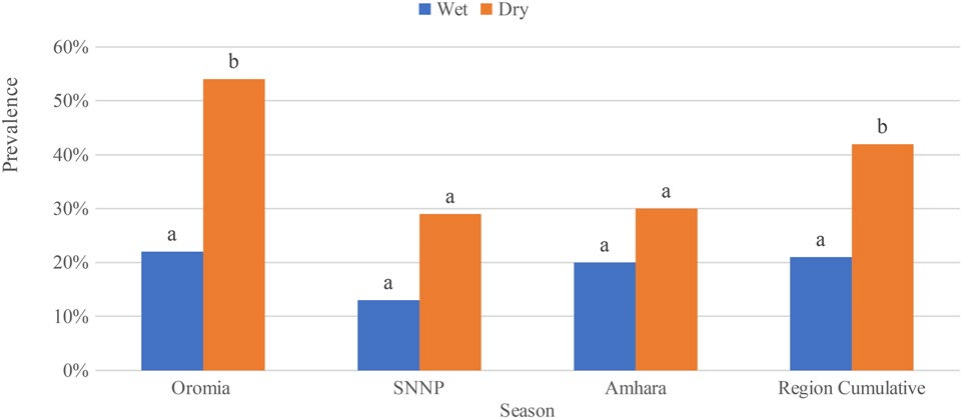
*Salmonella* prevalence in the raw milk samples collected from three regions in the dry and wet. Different letters above bars indicate significant differences at *P* < 0.05

**Fig. 3:**
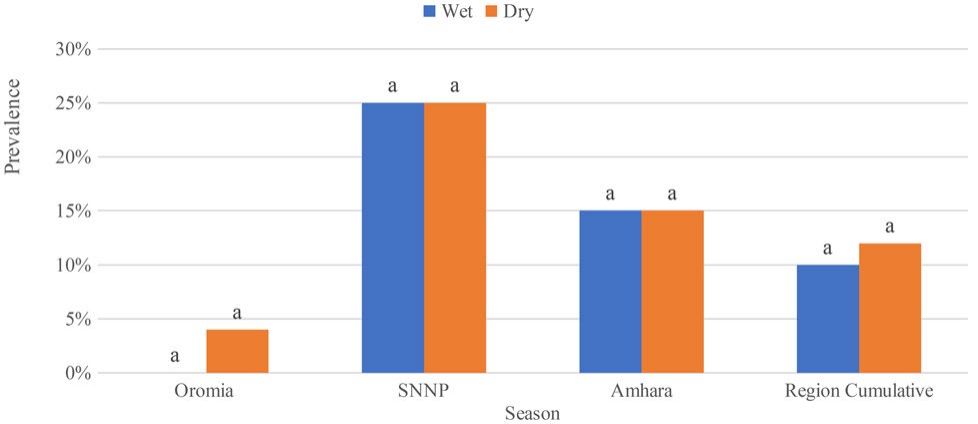
*Salmonella* prevalence in the pasteurized milk samples collected from three regions in the dry and wet. Different letters above bars indicate significant differences at *P* < 0.05

The overall prevalence of *S.enterica*for all sample types (raw milk, pasteurized milk, and cottage cheese), collected from all three study sites, during the wet season was 13.2%. Raw milk samples collected during the wet season had a significantly higher prevalence of *S.enterica*when compared to pasteurized milk and cottage cheese samples (χ2 = 11.41, *P* = 0.003). Likewise, during dry season, *S.enterica* was significantly recovered more from raw milk (χ2 = 34.53, *P* = 0.000)… Among the study regions, the samples collected in Oromia during the wet season had a significantly higher *S.enterica* prevalence as compared to Sidama and Amhara regions (Table 1). The variation in *S.enterica*prevalence is attributed to a higher detection rate of *S.enterica* from raw milk (χ2 = 18.72, *P* = 0.000) when compared to pasteurized milk and cottage cheese in Oromia region. This was the case also in the dry season (χ2 = 45.1, *P* = 0.000), 92.85% (*n* = 26) of tested raw milk samples tested positive for *S.enterica*. In Sidama, during wet season the prevalence of *S.enterica*was higher in pasteurized milk 3% (*n* = 6), but not significantly higher compared to raw milk and cottage cheese as shown in (Table 1). Even though the pattern detection of *S.enterica* was higher in raw milk 29.2% (*n* = 7) and pasteurized milk 25% (*n* = 6) in the dry season compared to cottage cheese 8.3% (*n* = 1), there was no significant variation (χ2 = 2.00, *P* = 0.367. The pattern of *S.enterica* prevalence in the Amhara region revealed that it was highly detected in raw milk 20% (*n* = 4) than in pasteurized milk 15% (*n* = 3) during the wet season but no significant variation has observed (Table 1). Similarly, during the dry season, it was recovered primarily from raw milk 25% (*n* = 5) and pasteurized milk 20% (*n* = 4), and no significant variation has been observed (χ2 = 1.77 *P* = 0.41).In terms of seasonal variation in the prevalence of *S.enterica*, we found significant differences in prevalence between wet and dry seasons only for raw milk samples collected from Oromia (χ2 = 7.3; *P* = 0.007; Fig. (2). In contrast, the differences were insignificant in Amhara and Sidama. Furthermore, no significant difference was found in cumulative *S.enterica* prevalence in pasteurized milk in three study sites (Fig. 3).

**Table 1:** Prevalence of *S. enterica* in tested dairy food sample types collected at different study sites in the wet season

Region	Sample Type	Number of Sample	Number of positive samples (%)	χ^2^	*P*-value
Oromia	Raw milk	48	13 (27.1)	**18.7**	0.000**
	Pasteurized milk	48	0 (8.8)		
	Cottage Cheese	24	1 (4.2)		
	**Total**	**120**	**14 (11.7)**		
SNNP	Raw milk	24	3 (12.5)	**4.1**	**0.13**
	Pasteurized milk	24	6 (25)		
	Cottage Cheese	12	0 (0)		
	**Total**	**60**	**9 (15)**		
Amhara	Raw milk	20	4 (20)	**1.8**	**0.399**
	Pasteurized milk	20	3 (15)		
	Cottage Cheese	8	0 (0)		
	**Total**	**48**	**7 (14.6)**		

^a^ SNNP, Southern Nation Nationalities and People. ^b^ χ^2^, Chi-square

### Comparison of overall prevalence of *S.enterica between dry and wet seasons across the value chain*

Significant differences in *S.enterica* prevalence in the wet season at different levels of the dairy value chains were found only in Oromia (χ2 = 19.31, *P* = 0.02) as shown in Table (2). The prevalence of *S.enterica* was similar at the producer 29% (*n* = 7) and collector 25% (*n* = 6) levels of the dairy value chain of raw milk. This finding is consistent with what was observed in the dry season, where the prevalence of *S.enterica* was found to be significantly varied (χ2 = 47.3, *P* = 0.00) among dairy value chains due to higher detection of *S.enterica* in producer 66,7% (*n* = 16) and collector 41.7% (*n* = 10) value chains of raw milk.

**Table 2:** Prevalence of *S. enterica* in wet season at different levels in the dairy value chain in three regions

Region	Level in the Value chain	Number of Sample	Number of positive samples (%)	χ^2^	*P*-value
**Oromia**	Producer	24	7 (29.2)	19.3	0.002
	Collector	24	6 (25)		
	Processor	24	0 (0)		
	Retailer	24	0 (0)		
	Farm market cottage cheese	12	0 (0)		
	Farm Retailer cottage cheese	12	1 (4.2)		
	**Total**	**120**	**14 (11.7)**		
**SNNP**	Producer	12	1 (8.3)	4.4	0.4
	Collector	12	2 (16.7)		
	Processor	12	3 (25)		
	Retailer	12	3 (25)		
	Farm market cottage cheese	6	0		
	Farm Retailer cottage cheese	6	0		
	**Total**	**60**	**9 (15)**		
**Amhara**	Producer	10	2 (20)	2.2	0.8
	Collector	10	2 (20)		
	Processor	10	2 (20)		
	Retailer	10	1 (5)		
	Farm market cottage cheese	4	0 (0)		
	Farm Retailer cottage cheese	4	0 (0)		
	**Total**	**48**	**7 (14.6)**		

^a^ SNNP, Southern Nation Nationalities and People. ^b^ χ^2^, Chi-square

In the Sidama region, *S.enterica* predominantly recovered from the processer 25% (*n* = 3) and retailer 25% (*n* = 3) of the value chain of pasteurized milk during the wet season but doesn’t significantly higher as mentioned in Table (2). Likewise, no significant variation has been observed in the dry season (χ2 = 8.3, *P* = 0.14) in *S.enterica* prevalence at different levels of the dairy value chain. Even though there was no significant variation in *S.enterica* prevalence in the dry season it was typically recovered from the collector 41.67% (*n* = 5) and processer 41.67% (*n* = 5) value chain.

The prevalence of *S.enterica* in the dairy value chain that was located in the Amhara region had no significant variation as Table (2) demonstrated. Nevertheless, producer 20% (*n* = 2) and collector 20% (*n* = 2) value chains of the raw milk and processer 20% (*n* = 2) value chain of pasteurized milk had relatively higher *S.enterica* detection during wet season. The detection of *S.enterica*during dry season in Amhara region was highly detected in the producer 40% ( *n* = 4) value chain of raw milk and retailer 30% (*n* = 3) value chain of pasteurized even though no significant variation has been observed (χ2 = 2.7, *P* = 0.74).

**Fig. 4:**
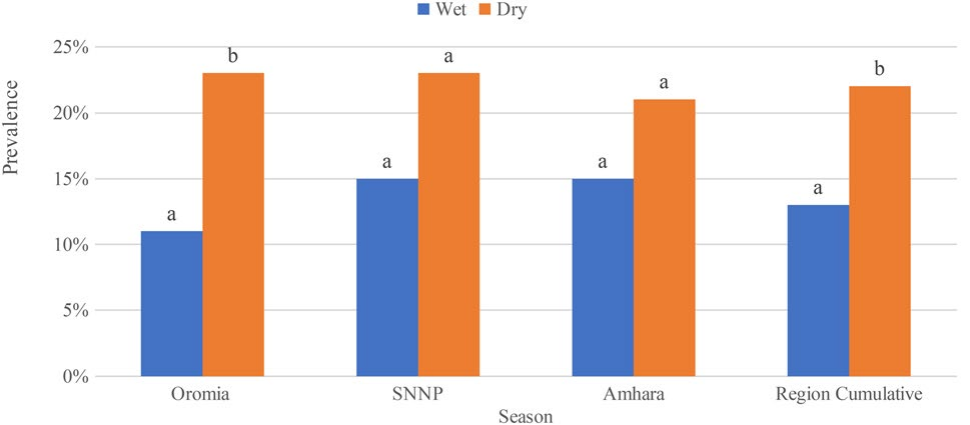
Cumulative prevalence of *Salmonella* in all samples type of the study collected from three regions in the dry and wet. Different letters above bars indicate significant differences at

**Fig. 5:**
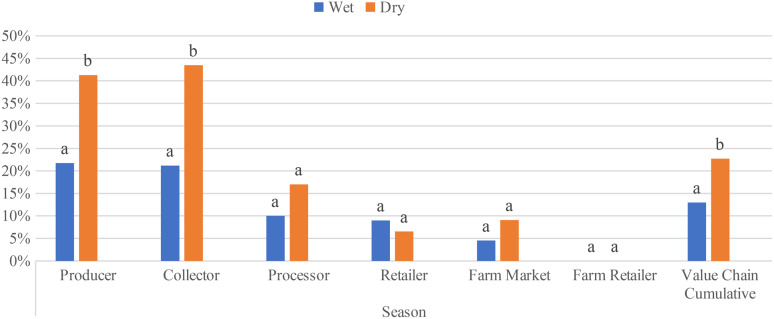
Cumulative prevalence of *Salmonella* samples collected from dairy value chains of the three study regions in the dry and wet season. Different letters above bars indicate significant differences at *P* < 0.05

Seasonality of *S.enterica* prevalence among dairy value chain only observed in the Oromia region, in which the detection of *S.enterica* was significantly higher during the dry season (χ2 = 5.7, *P* = 0.017) (Fig. 4). This variation comes only from producer value chain (χ2 = 5.4, *P* = 0.02) than collector (χ2 = 2.3, *P* = 0.13), processor (χ2 = 2.09, *P* = 0.15) and farm market (χ2 = 1.0, *P* = 0.31); whereas in Sidama (χ2 = 1.345, *P* = 0.294) and Amhara (χ2 = 0.643, *P* = 0.425) no significant difference has been observed in *S.enterica* prevalence during dry and wet seasons. Fig. 5)

## Discussion

The ability of *S.enterica* to resist environmental stress as well as proliferate is enhanced when temperature increases (Montville et al., 2012). Almashhadany et al. (2019) reported that the detection of the *S.enterica*in cow milk samples gradually increased with r² =0.854 value in the spring season and early summer; the prevalence of the *S.enterica* was 19.51% (*n* = 41) in June and May and 2.4% (*n* = 41) in January and February. Pangloli et al. (2008) reported that contamination of *S.enterica*in raw milk found to be lower in winter (June-March) (*n* = 24, 6%) and fall (October-December) (*n* = 24, 7%) than in spring (April-June) (*n* = 24, 17%) and summer (July-September) (*n* = 24, 29%). However, Gebeyehu et al. (2022) found the rate of detection of bacteria in raw milk samples was higher in the wet season 11.98% (*n* = 192) than in the dry season 8.85% (*n* = 192). They also reported that even though the detection rate of bacteria was higher in the wet season, it wasn’t significantly different from that of the dry season, which supports the current findings in Sidama and Amhara regions for the raw milk *S.enterica* seasonal prevalence.

The previous *S.enterica* prevalence reports from the Oromia region by Abunna et al. (2017) (Mojo) revealed a lower prevalence, but Mossie and Dires (2016) (Debre Zeit) reported a (23.5%) higher prevalence compared to the wet season prevalence of *S.enterica*. The higher prevalence could be attributed to the study’s large sample size and the fact that the samples were collected during the dry season. In the case of Sidama region, Madalcho et al. (2019) (Wolayta), Fesseha et al. (2020) (Hawassa), and Belay et al. (2022) (Gamo Zone) have reported 1.8%, 3.3%, and 8.3% prevalence, respectively, which was lower than the current study finding of wet season *S.enterica* in the SIDAMA region. While Senbetu (2014) and Habtamu et al. (2018) from Hawassa reported a higher prevalence rate of *S. enterica*, 25%, and 17.8%, respectively. Among the *S.enterica* prevalence reports from the Amhara region, Abebe et al. (2023) (Dessie and Kombolcha), Mulaw (2017), and Hailu et al. (2015) (Gondor) reported 5.92%, 9.35%, and 3.24%, respectively, which is lower than the *S.enterica* prevalence report during the wet season.

Several studies have revealed the importance of milk and dairy products in the transmission of *S.enteric a*and the development of salmonellosis in humans due to seasonal fluctuation (Mazurek et al. 2004; Dominguez et al. 2009; van Duynhoven et al. 2009; Giacometti et al. 2015; Putturu et al. 2015; Ung et al. 2019). Stephen and Barnett (2016) reported that the standard regression model estimated salmonellosis (59.4%) cases were increased when there was a 5 °C increase in mean temperature. Zhang et al. (2010) reported a 62% increase with a 5 °C mean temperature change. Similarly, Judd et al. (2019) (USA) found that the overall case count of salmonellosis became higher in the summer (38.6%) and lower in the winter (14.5%).

The impact of seasonal fluctuation on *S.enterica* detection in food items other than dairy products revealed the prevalence predominantly recovered from the samples that were collected in the warmer season (Lal et al. 2012). Calle et al. (2021) reported the contamination of *S.enterica* on beef carcasses was found to be higher in samples collected during the dry season (*n* = 103, 6%) than those collected during the rainy season (*n* = 102, 1.96%) with (OR 5.90, 95% CI 1.18–29.57) value. Barkocy-Gallagher et al. (2003) revealed that the detection of *S.enterica* from evisceration of beef carcasses was higher in summer and fall than in the winter and spring.

The super-shedding phenomena and changes in animal feeding habits brought on by climate change can both affect the prevalence and shedding rates of infection agents (Williams et al. 2015; Smith et al.,2019). When temperature is higher, cattle may graze outside more frequently. This can increase pathogen survival and shedding rates, and lactating cows infected with *S.enterica* may contaminate milk if proper hygiene practices aren’t followed (Jacob et al. 2009). There is evidence for the higher detection rate of the *S.enterica* in the dry (Sunny) season than wet (rainy) season. Fosseler et al. (2005) found that seasonal impacts of season on the detection of the *S.enterica* in the fecal materials of the cows revealed that the bacterium was detected at higher rates in summer (OD = 2.7) and spring (OD = 2.3) and slightly higher in fall (OD = 2.1) than winter (OD = 1). In addition, Pangloli et al. (2008) reported the *S.enterica* predominantly detected in those lactating cow facial samples that were gathered in the summer (72%) rather than in the fall (27%) and winter (13%). The recovery of the *S.enterica* was also 3.49-fold (Mental-Haenszel’s weighted odd ratio) higher during the months of May-July compared to February-April according to Wells et al. (2001).

## Conclusion

In this study, we showed that *S.enterica* prevalence varied with the seasons, geographic locations, and dairy product type. In comparison to the wet seasons, the prevalence of *Salmonella* was notably higher during the dry season. In dry and wet seasons, samples of raw milk taken at the production stage were substantially more contaminated by *S.enterica* than pasteurized milk and cottage cheese, which justifies thorough heat treatment before consuming raw milk. Additionally, it was alarming to find that the pasteurized samples obtained from processor gates and retailers were tainted and required stringent intervention by means of enforcement of the implementation of Hazard Analysis and Critical Control Points (HACCP) and thorough follow-up by regulatory bodies for consumer health. The best way to ensure sustainable interventions is through the application of behaviour change communication training on good hygienic practices (GHP) and good agricultural practices (GAP) by identifying the natural ecosystems that contribute to contamination in order to ensure the microbiological safety of milk and cottage cheese.

### Electronic supplementary material

Below is the link to the electronic supplementary material.

Supplementary Material 1

Supplementary Material 2

Supplementary Material 3

## Data Availability

Not applicable.
